# Potential mechanism of the Shunaoxin pill for preventing cognitive impairment in type 2 diabetes mellitus

**DOI:** 10.3389/fneur.2022.977953

**Published:** 2022-10-21

**Authors:** Yuejie Guo, Ning Luo, Xueran Kang

**Affiliations:** ^1^Department of Geriatrics, The First People's Hospital of Chenzhou, Chenzhou, China; ^2^Department of Endocrinology, The First People's Hospital of Chenzhou, Chenzhou, China; ^3^Shanghai Jiao Tong University College of Basic Sciences, Shanghai, China

**Keywords:** Shunaoxin pill, diabetes, cognitive impairment, network pharmacology, molecular docking

## Abstract

**Objective:**

This study aims to analyze the efficacy and mechanism of action of the Shunaoxin pill in preventing cognitive impairment in diabetic patients using network pharmacology.

**Methods:**

The main active compounds of the Shunaoxin pills and their action targets were identified *via* the TCMSP and Batman-TCM databases. The GEO database was used to identify the genes in type 2 diabetic individuals associated with cognitive impairment. Subsequently, a common target protein-protein interaction (PPI) network was constructed using the STRING database, and targets associated with diabetes and cognitive impairment were screened by performing a topological analysis of the PPI network. The AutoDock Vina software was used for molecular docking to evaluate the reliability of the bioinformatic analysis predictions and validate the interactions between the active ingredients of the Shunaoxin pill and proteins associated with diabetes and cognitive impairment.

**Results:**

Based on the TCMSP and Batman-Tcm platform, 48 active ingredients of the Shunaoxin pill were identified, corresponding to 222 potential action targets. Further analysis revealed that 18 active components of the Shunaoxin pill might contribute to cognitive impairment in type 2 diabetic patients. Molecular docking simulations demonstrated that the active ingredients of the Shunaoxin pill (hexadecanoic acid, stigmasterol, beta-sitosterol, and angelicin) targeted four core proteins: OPRK1, GABRA5, GABRP, and SCN3B.

**Conclusion:**

Active ingredients of the Shunaoxin pill may alleviate cognitive impairment in diabetic patients by targeting the proteins OPRK1, GABRA5, GABRP, and SCN3B.

## Introduction

Diabetes mellitus (DM) is a metabolic disorder characterized by hyperglycemia caused by genetic and environmental factors and is the third most common non-communicable disease (1–4). DM has become a major health problem worldwide owing to its increasing prevalence and associated disability and mortality (5–7). The global prevalence of diabetes is expected to increase to 7.7% by 2030 (8, 9), and ~592 million people are expected to suffer from diabetes by 2035 (10). Furthermore, diabetic complications also increase dramatically as the incidence of diabetes increases (11). Due to inadequate insulin secretion or insulin resistance, T2DM patients experience hyperglycemia, leading to chronic damage to blood vessels, neurons, brains, and other organs (12, 13). Moreover, diabetes may also cause nervous system complications, such as cognitive impairment (14, 15). Mild cognitive impairment is estimated to affect 45% of type 2 diabetes patients (16). Learning and memory impairment are the most distinctive features of cognitive impairment in DM (17, 18). Diabetic patients may suffer from cognitive impairment, leading to abnormalities in brain neuroplasticity and energy metabolism (19). DM is associated with cognitive impairment in the elderly, a risk factor for dementia, including Alzheimer's disease (AD) (20, 21). Cognitive impairment may also result in brain deterioration or neurodegenerative diseases (16, 22). In addition, it is estimated that diabetes causes ~10–15% of dementia cases, with patients demonstrating poorer self-management skills and glycemic control (16, 23). The healthcare costs for dementia patients are 1.5 times higher than control subjects of the same age group without dementia (24), and the financial figures indicate a significant burden for patients with cognitive impairment and society. However, the pathophysiology of diabetes-induced cognitive impairment remains poorly understood, and timely and adequate diagnostic and therapeutic tools are still lacking.

Chinese medicines have multi-faceted and multi-channel effects (25–28). Network pharmacology enables the exploration of the active ingredients and potential targets of Chinese medicine by providing a holistic view of complex systems interacting with multiple disease targets (29, 30). As a result, network pharmacology has gained popularity for studying key disease-related targets and biological functions and predicting potential synergistic mechanisms against complex diseases (31, 32). Shunaoxin pills, a drug with significant therapeutic potential, have been shown to be able to dilate the thoracic aorta of isolated rats (33). A clinical study investigating the effects of the Shunaoxin pill has reported an alleviation in diabetes-induced cognitive decline (34). The Shunaoxin pill consists of two herbs, Chuanxiong and Angelica, both of which can improve chronic cerebral ischemia (35). Researchers have found that the chemical components of the Shunaoxin pill, including ferulic acid and ligustilide, possess hypoglycemic, antioxidant, and anti-inflammatory properties (36–38). Furthermore, the Shunaoxin pill can be used to treat a variety of cardiovascular and cerebrovascular diseases, such as cerebral ischemia and inadequate blood supply to the vertebral basilar artery (39). The mechanisms of cognitive impairment vary across different types of diabetes. Cognitive impairment in type 1 diabetes is associated with persistent hyperglycemic states, diabetic ketoacidosis, and hypoglycemic episodes (40, 41). On imaging, type 2 diabetes mellitus (T2DM) patients with cognitive impairment often exhibit cerebral vasculopathy, cortical atrophy, and hippocampal volume reduction (18, 42). In addition, neuroinflammation and oxidative stress are often observed in diabetes-related cognitive impairment (43–47). Therefore, the Shunaoxin pill can somewhat alleviate diabetes-related cognitive impairment. However, it is still unclear how the Shunaoxin pill prevents cognitive impairment in diabetics.

This study applied network pharmacology and molecular docking techniques to determine Shunaoxin's mechanism of action in improving cognitive impairment in diabetic patients. The study aimed to provide scientific support for traditional Chinese medicine as a treatment for cognitive impairment in diabetics from a modern medical perspective.

## Materials and methods

### Screening of active ingredients and drugs' targets

The active ingredients of Chuanxiong Rhizoma, Angelicae Sinensis Radix, and the drug composition of the Shunaoxin pill were searched on the TCMSP with the following screening conditions: oral bioavailability (OB) ≥30%, drug-likeness (DL) ≥0.18, and half-life (HL) ≥4. The active ingredients of Chuanxiong Rhizoma and Angelicae Sinensis Radix were searched in the Batman-Tcm database (http://bionet.ncpsb.org.cn/batman-tcm/) to obtain potential targets. The score cutoff was set to 100, and the adjusted *P*_value cutoff was set to 0.05. Subsequently, the Uniprot database (https://www.uniprot.org/) was used to identify the gene names of the matching targets (48, 49).

### Disease target acquisition

The gene microarray data related to “cognitive impairment in type 2 diabetes” was downloaded from the GEO (gene expression omnibus) database (https://www.ncbi.nlm.nih.gov/geo/). The study data were obtained from 19 healthy adults and 17 patients with type 2 diabetes from the microarray dataset GSE138260, 15 patients with Alzheimer's disease, and 18 patients with Alzheimer's disease combined with type 2 diabetes from the microarray dataset GSE161355. Microarray data background correction, normalization, and expression value calculation were performed using the Bioconductor R package in R software. The limma package was used to calculate the differentially expressed mRNAs between the two groups. The screening criteria for differential genes were set as *P* < 0.05 and expression change ≥1.5-fold (|log_2_ FC|≥0.58). Upregulated mRNA expression was defined as log_2_FC ≥0.58, and down-regulated mRNA expression was defined as log2FC ≤−0.58. Finally, the differentially expressed genes (DEGs) for cognitive impairment in type 2 diabetic patients were finally derived. The heatmap package was used for cluster analysis of the screened DEGs, and the *P* of the differentially expressed data were transformed to –log10, which was grouped according to log_2_ FC.

### Common target screening of active ingredients and diseases and PPI network construction

The R software (https://www.r-project.org/) and Perl programs were used to identify the intersection between disease targets and drug targets, and the Venn diagram was generated by the Venny 2.1 software (http://bioinfogp.cnb.csic.es/tools/venny/index.html). The protein–protein interaction (PPI) was constructed using the STRING database (https://string-db.org/). The protein species was set to “Homo sapiens,” medium confidence was set to 0.4, and other parameters were kept to the default settings. The potential protein-protein interaction network (PPI network) was obtained by Cytoscape 3.7.2 software (https://cytoscape.org/), and topological analysis of the PPI network was performed to screen the key targets.

### Gene ontology biofunctional analysis and Kyoto encyclopedia of genes and genomes pathway enrichment

The clusterProfilerGO R package was used to analyze the common targets of the Shunaoxin pill active ingredients (50). Gene ontology (GO) analysis is mainly used to characterize the functions of gene products, including cellular components (CC), molecular functions (MF), and biological processes (BP). The Kyoto encyclopedia of genes and genomes (KEGG) pathway enrichment analysis was also performed by applying the clusterProfilerKEGG package, while the corresponding signaling pathways were mapped using the path view package. Moreover, the degree of core pathway enrichment was analyzed to investigate the possible biological functions and signaling pathway mechanisms of the active ingredients of the Shunaoxin pill in the treatment of cognitive impairment in type 2 diabetic patients based on the enrichment factor values.

### Molecular docking verification

The interactions between the top four core active ingredients and the core proteins obtained from the preliminary network pharmacology screening were validated by molecular screening. The structural formula of the active ingredient was downloaded from the PubChem database (https://pubchem.ncbi.nlm.nih.gov/), and the corresponding 3D structure was produced with Chem3D software (51). The PDB format of the core protein structural domain was then downloaded from the PDB database (http://www.rcsb.org/), and protein dehydration and dephosphorylation were performed by PyMOL software. The active drug ingredient and core protein gene file were converted from PDB format to PDBQT format by the AutoDockTools 1.5.6 software, and the active pockets were identified. Finally, the Vina script was run to calculate the molecular binding energy and molecular docking results, while docking sites were identified with Discovery Studio 2019, and the LibDockScore was calculated for flexible binding. The molecular docking results were imported into PyMOL software for molecular docking conformation display. The binding energy of <0 indicates spontaneous binding of the ligand and the receptor. For the results showing Vina binding energy ≤−5.0 kcal/mol and LibDockScore >100, the ligand-receptor complexes were examined in 3D and 2D to evaluate the reliability of the bioinformatics predictions as per previous research (52–55).

## Results

### Active drug ingredients and corresponding targets

**Figure 1:**
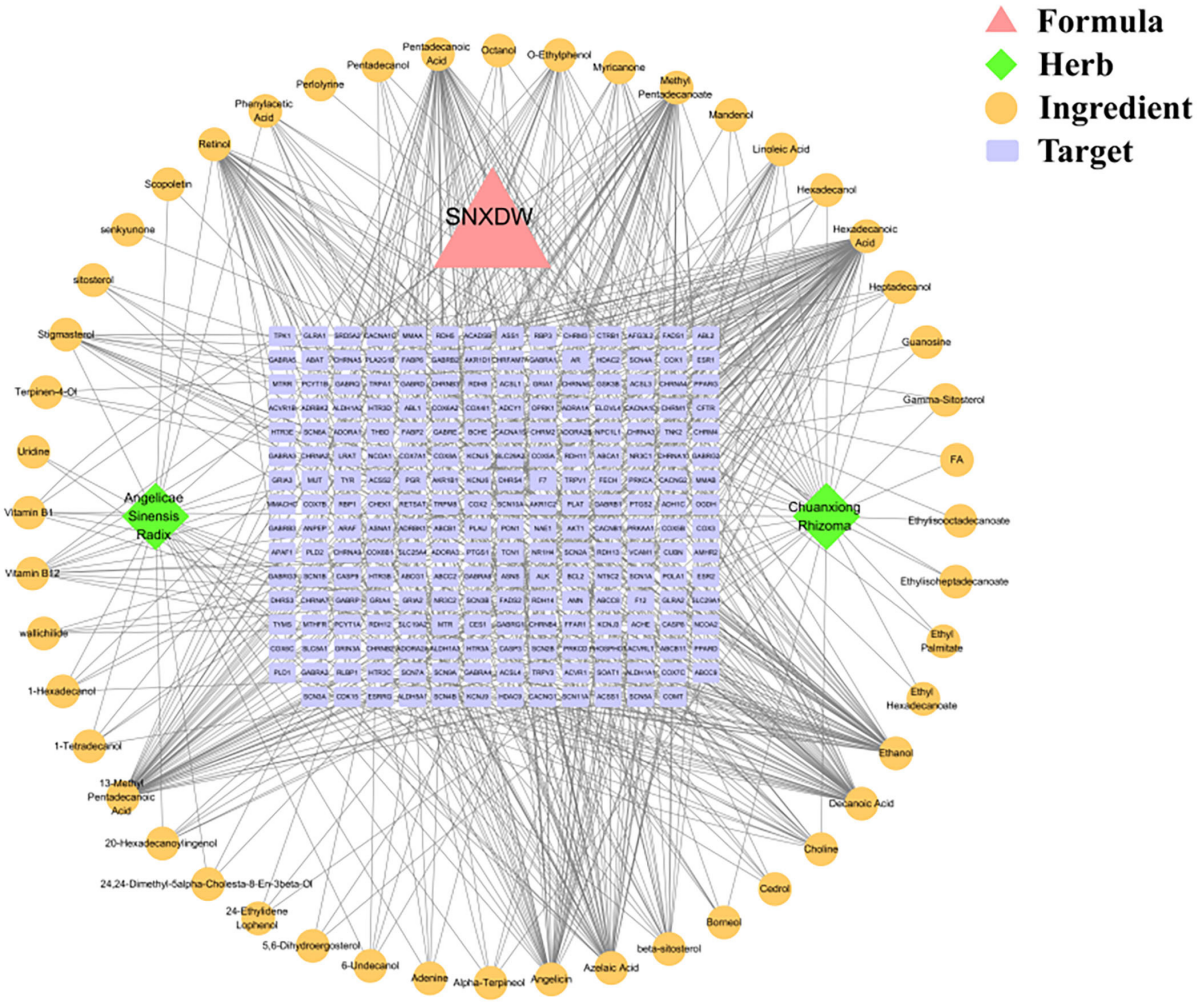
Active ingredients of the Shunaoxin pill (SNXDW)-target gene PPI network.

The relevant action targets of the Shunaoxin pill active ingredients were obtained from the TCMSP database, and the Uniprot database (https://www.uniprot.org/) was used to correct the matching target gene names. Finally, 48 active ingredients were obtained, corresponding to 222 potential targets. Cytoscape 3.7.2 software was used to construct the topology of the Shunaoxin pill active ingredient target network (Figure 1). Eight active ingredients were identified, namely hexadecanoic acid, ethanol, 13-methyl pentadecanoic acid, methyl pentadecanoate, pentadecanoic acid, azelaic acid, decanoic acid, angelicin. These may be the main active ingredients in Shunaoxin pills.

### Screening for disease targets

**Figure 2:**
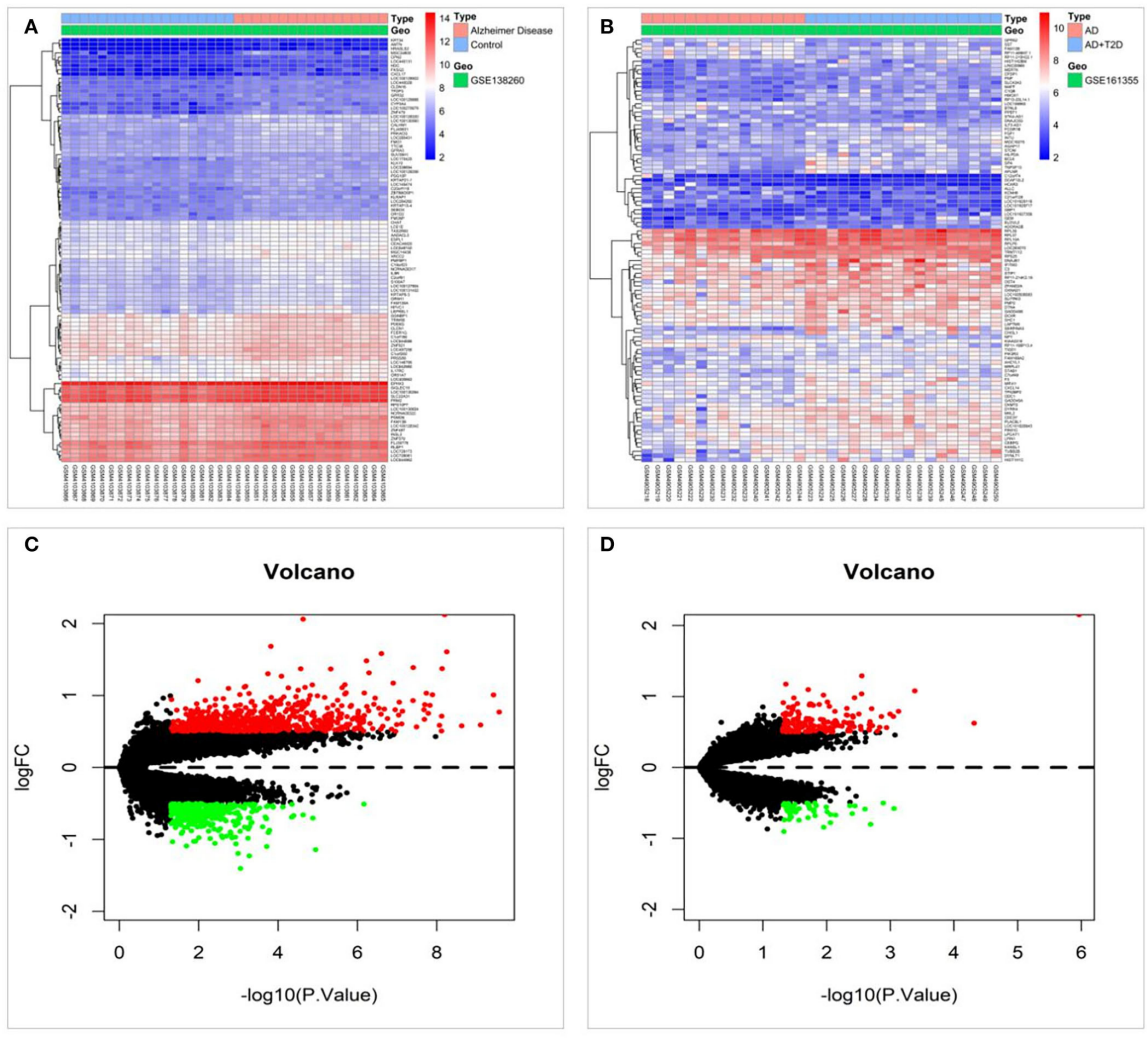
Differentially expressed mRNAs were screened in the GSE138260 and GSE161355 datasets. **(A)** Heat map of differentially expressed genes in the GSE138260 dataset. **(B)** Heat map of differentially expressed genes in the GSE161355 dataset. **(C)** Volcano plot of differentially expressed genes in the GSE138260 dataset. **(D)** Volcano plot of differentially expressed genes in the GSE161355 dataset.

To screen for protein targets associated with diabetes and cognitive impairment, 993 differentially expressed mRNAs were screened in the GSE138260 dataset, including 652 upregulated and 341 downregulated mRNAs. The GSE161355 dataset screened 196 differentially expressed mRNAs, including 145 upregulated and 51 downregulated mRNAs. Figures 2A,B display the heat maps based on the *p*-value screening of the top 100 most significant DEGs. Those processed data were imported into R to generate volcano plots, as shown in Figures 2C,D. Diabetes and cognitive impairment may be associated with these differential mRNAs.

### Common target screening and interaction network construction

**Figure 3:**
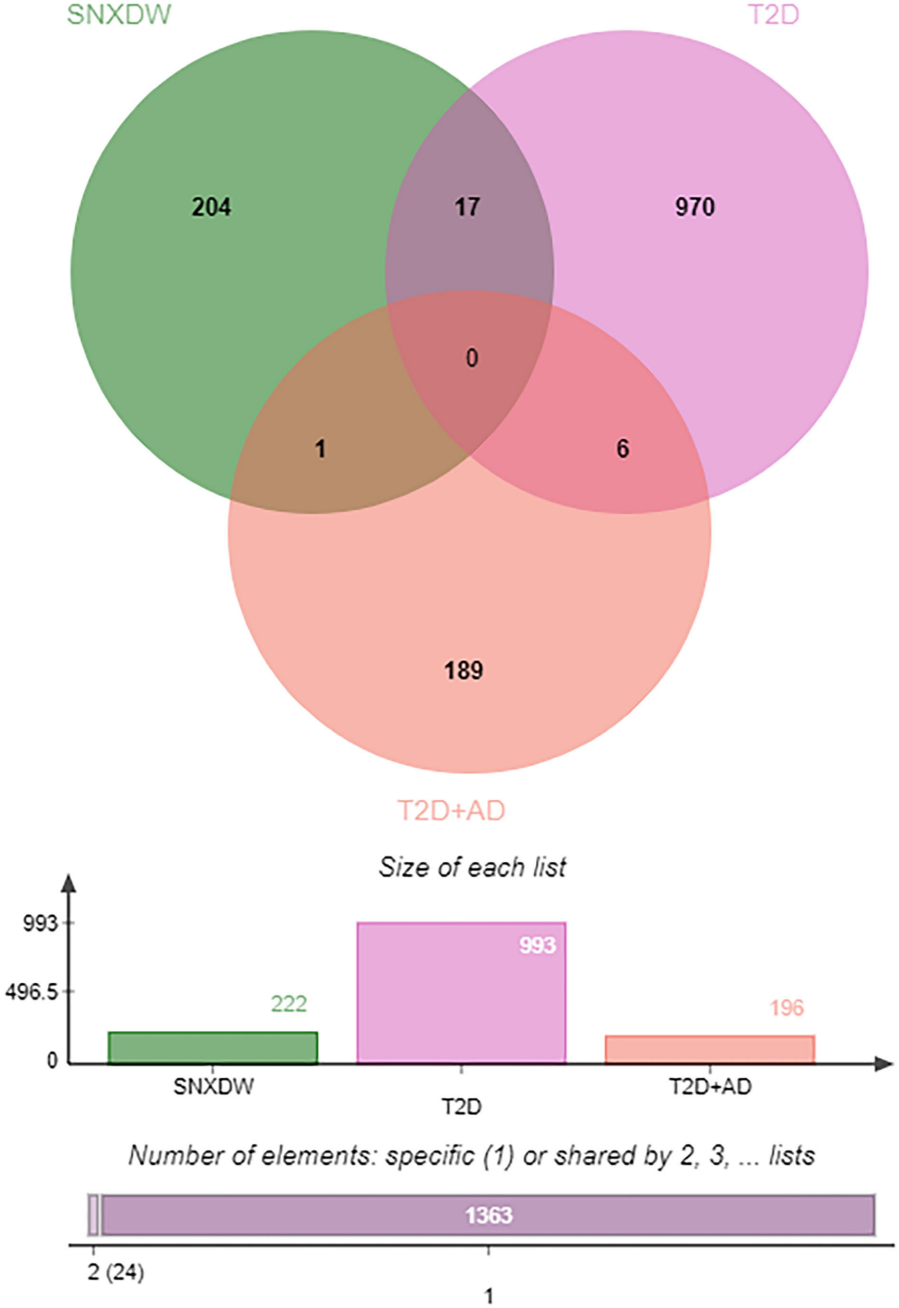
Venn diagram of drug active ingredient-disease intersection targets.

**Figure 4:**
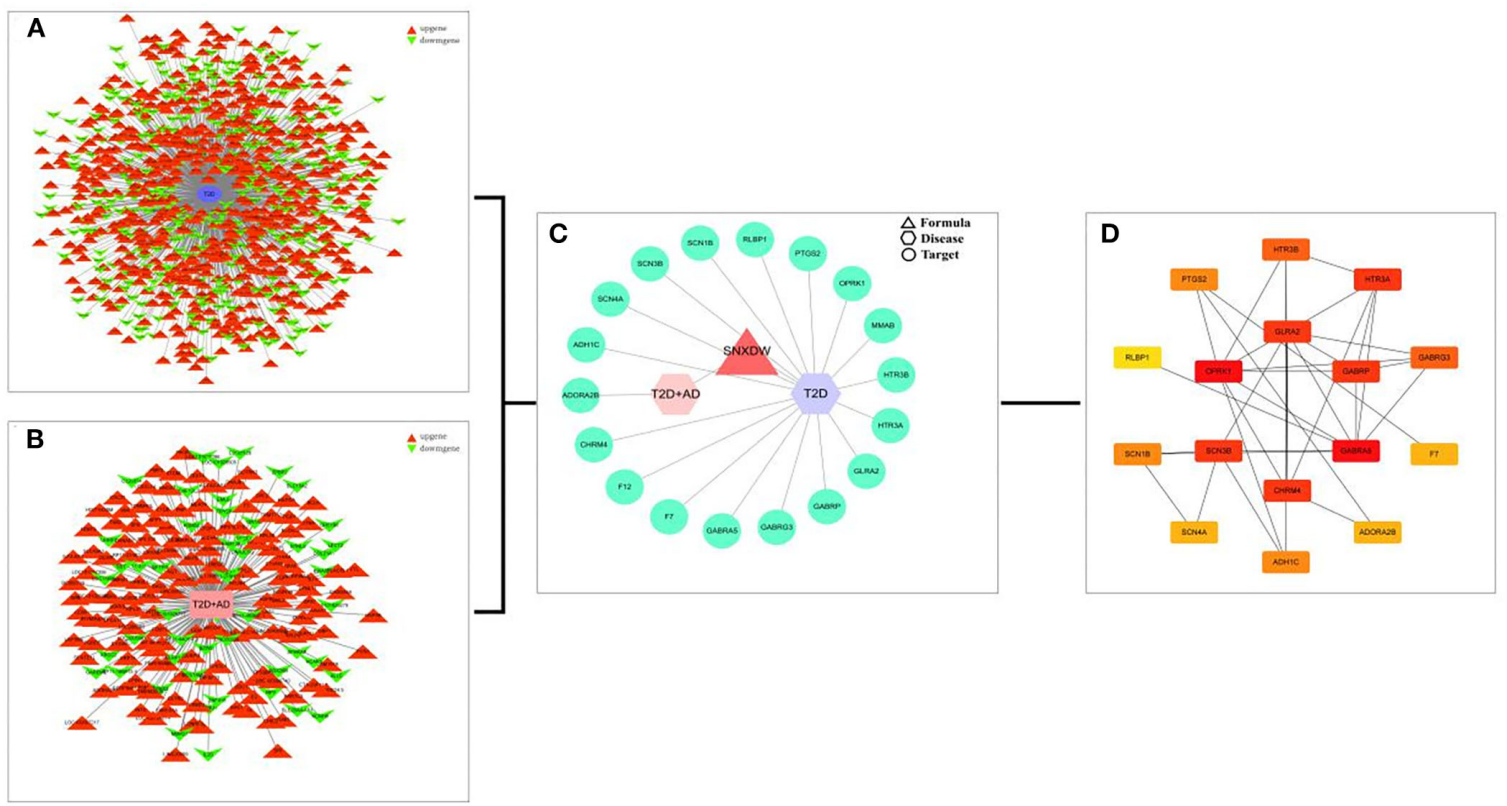
Interaction networks. **(A)** GSE138260 differentially expressed genes interaction network. **(B)** GSE161355 differentially expressed gene interaction network. **(C)** PPI network was constructed by Shunaoxin pill-disease-target genes. **(D)** Core 16 potential target genes were calculated based on degree values.

All active ingredient targets of the Shunaoxin pill, the GSE138260 dataset, and the GSE161355 dataset were imported into the online Venn diagram production site jvenn, and 18 intersecting potential targets of action were obtained (Figure 3). Cytoscape 3.7.2 was used to construct the target network for cognitive impairment in type 2 diabetics (Figures 4A,B). The intersecting target genes were imported into the STRING online analysis website (https://string-db.org/), and the protein-protein interaction results were exported (Figure 4C). After that, the CytoHubba plugin was used to obtain the core 16 potential target genes based on the degree algorithm as in previous research (Figure 4D) (56). Therefore, the Shunaoxin pill may target proteins that have been identified as targets as a result of these screenings.

### GO and KEGG enrichment analysis results

**Figure 5:**
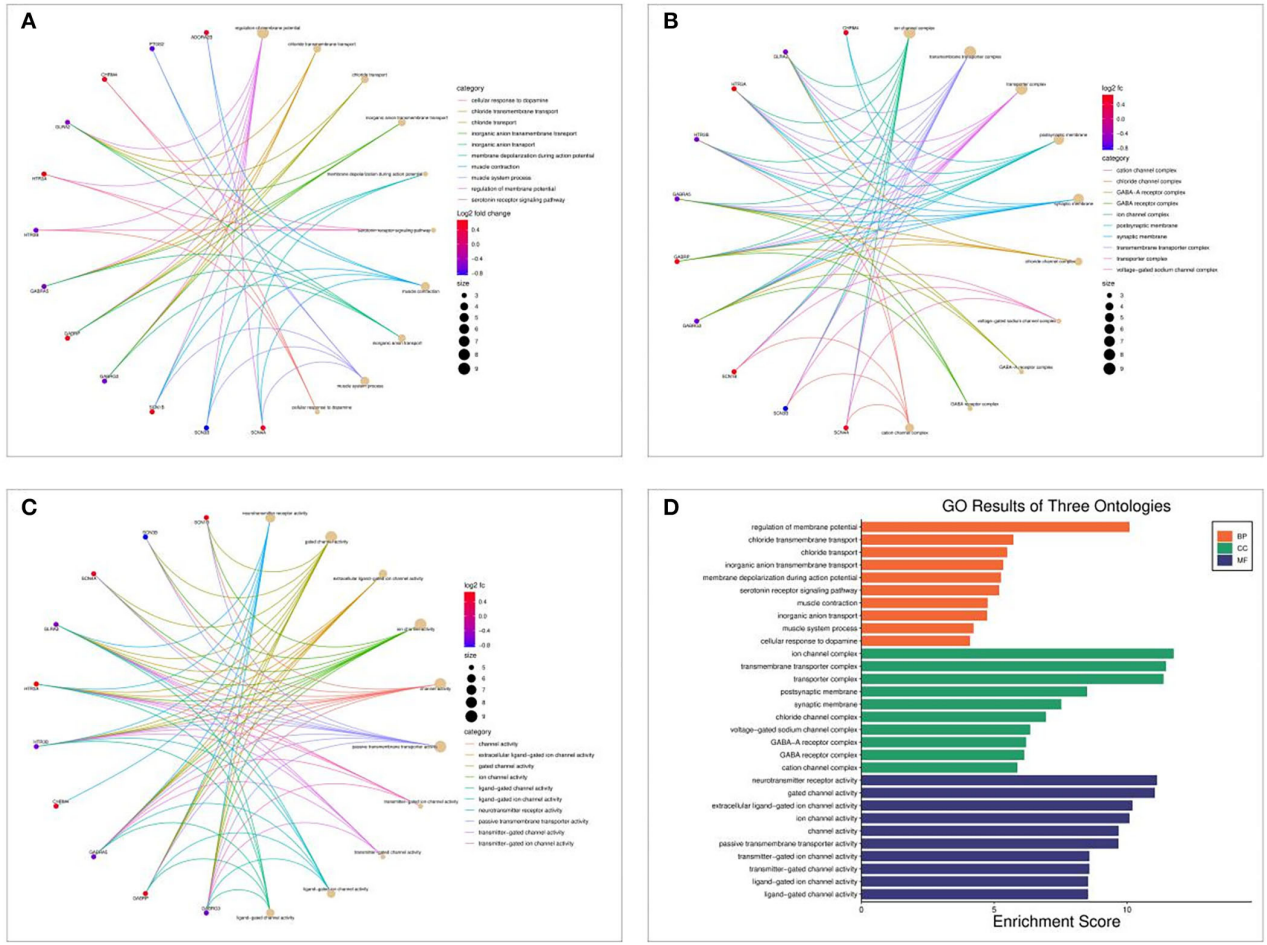
GO enrichment analysis of 18 potential targets of Shunaoxin pill for the treatment of cognitive impairment in patients with type 2 diabetes. **(A)** BP functional analysis chord diagram. **(B)** CC functional analysis chord diagram. **(C)** MF functional analysis chord diagram. **(D)** GO enrichment analysis histogram.

The Bioconductor package and cluster profile package in R were used to analyze GO and KEGG pathway enrichment analysis of Shunaoxin pill target proteins. GO analysis revealed that the 18 potential targets were mainly enriched in the biological processes of membrane potential regulation, chloride transmembrane transport, chloride transport, etc (Figure 5A). In addition, cell composition (CC) was mainly enriched in ion channel complexes, transmembrane transporter complexes, transporter complexes, etc (Figure 5B); molecular function (MF) was enriched primarily in neurotransmitter receptor activity, gated channel activity, extracellular ligand-gated ion channel activity, etc (Figure 5C). These GO enrichment pathways are shown in Figure 5D.

**Figure 6:**
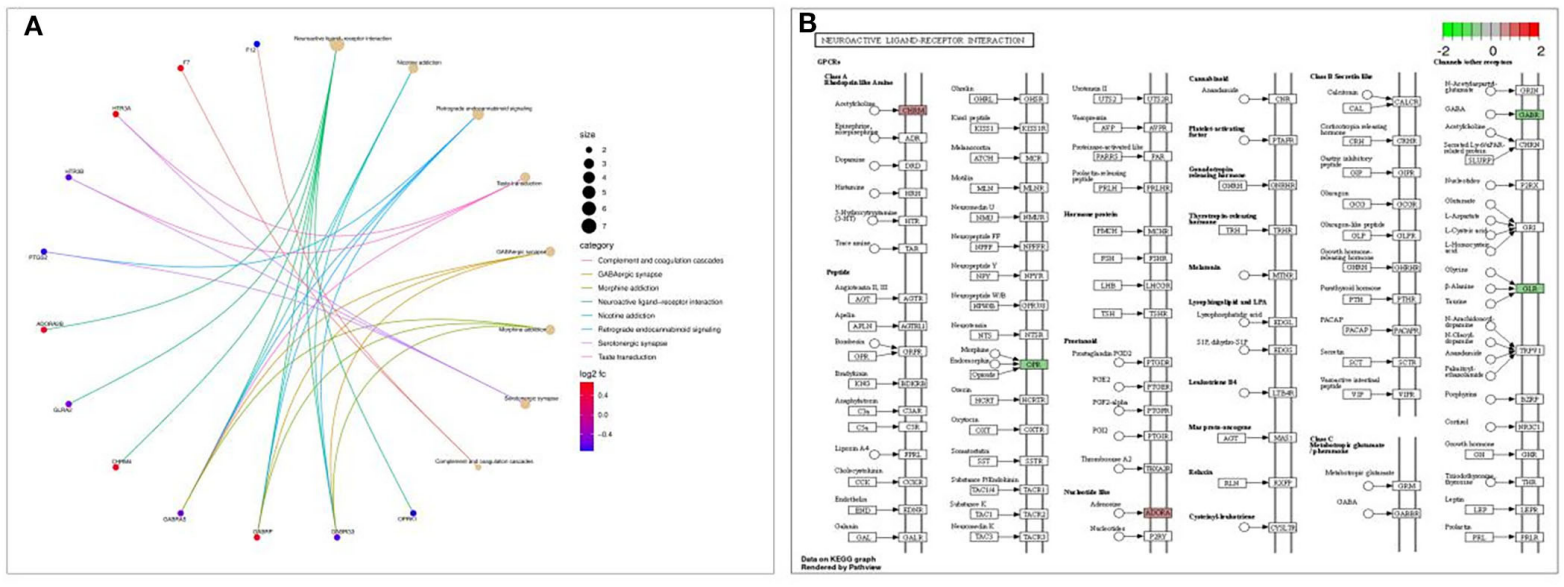
Enrichment analysis. **(A)** KEGG enrichment analysis bubble diagram. **(B)** Neuroactive ligand-receptor interaction signaling pathway.

KEGG pathway enrichment analysis revealed that it was mainly concentrated in inflammatory signaling pathways such as neuroactive ligand-receptor interaction, nicotine addiction, retrograde endocannabinoid signaling, taste transduction, and GABAergic synapse (Figure 6A). The path view package in R software was used to display the signaling pathway map associated with cognitive impairment in Shunaoxin pill-treated patients with type 2 diabetes (Figure 6B). The neuroactive ligand-receptor interaction signaling pathway was found to be enriched in the potential target proteins of the Shunaoxin pill.

### Results of molecular docking

**Figure 7:**
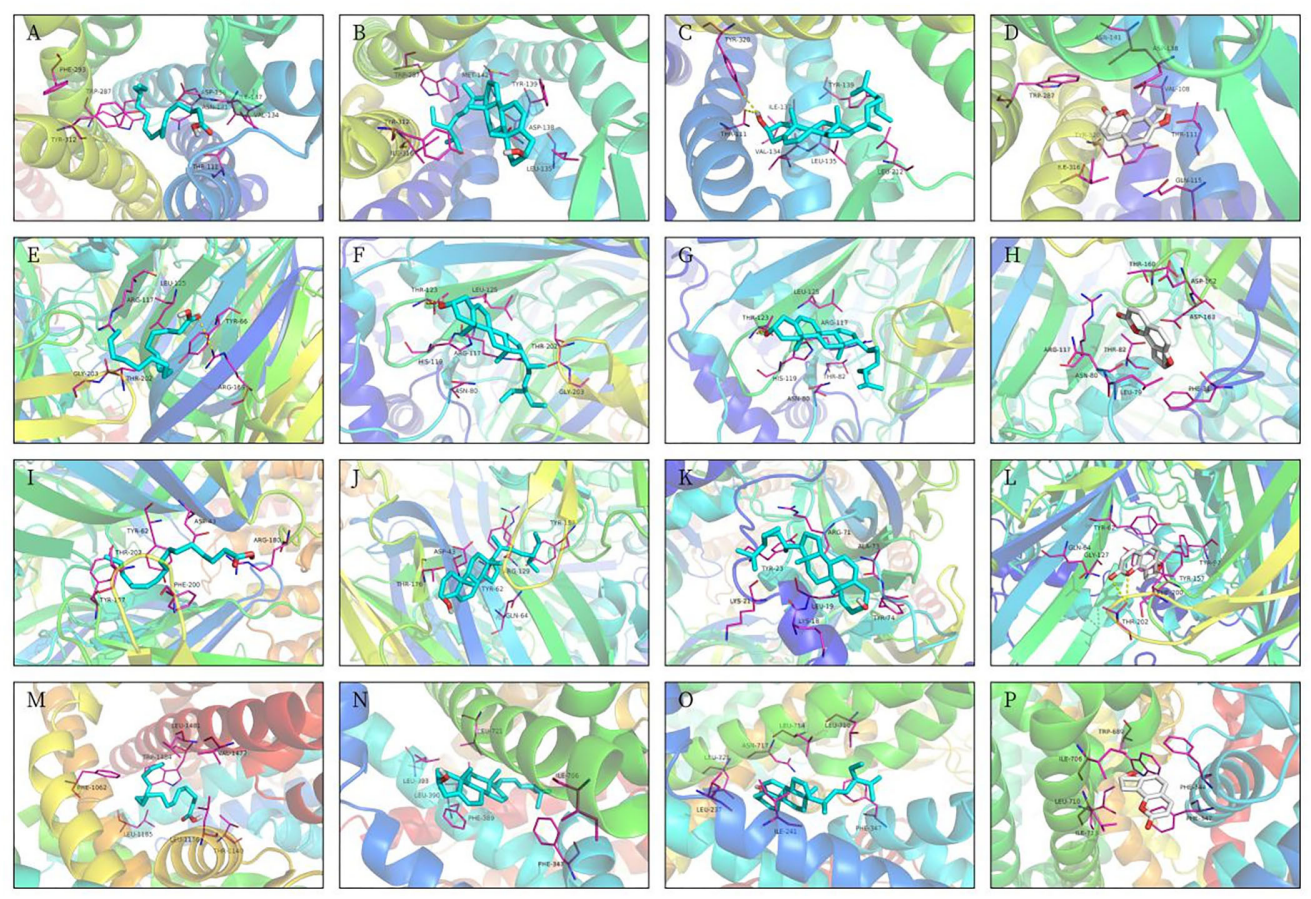
Molecular docking 3D models. **(A)** OPRK1-hexadecanoic acid complex. **(B)** OPRK1-stigmasterol complex. **(C)** OPRK1-beta-sitosterol complex. **(D)** OPRK1-angelicin complex. **(E)** GABRA5-Hexadecanoic acid complex. **(F)** GABRA5-stigmasterol complex. **(G)** GABRA5-beta-sitosterol complex. **(H)** GABRA5-angelicin complex. **(I)** GABRP-hexadecanoic acid complex. **(J)** GABRP-stigmasterol complex. **(K)** GABRP-beta-sitosterol complex. **(L)** GABRP-angelicin complex. **(M)** SCN3B-Hexadecanoic acid complex. **(N)** SCN3B-stigmasterol complex. **(O)** SCN3B-beta-sitosterol complex. **(P)** SCN3B-angelicin complex.

**Figure 8:**
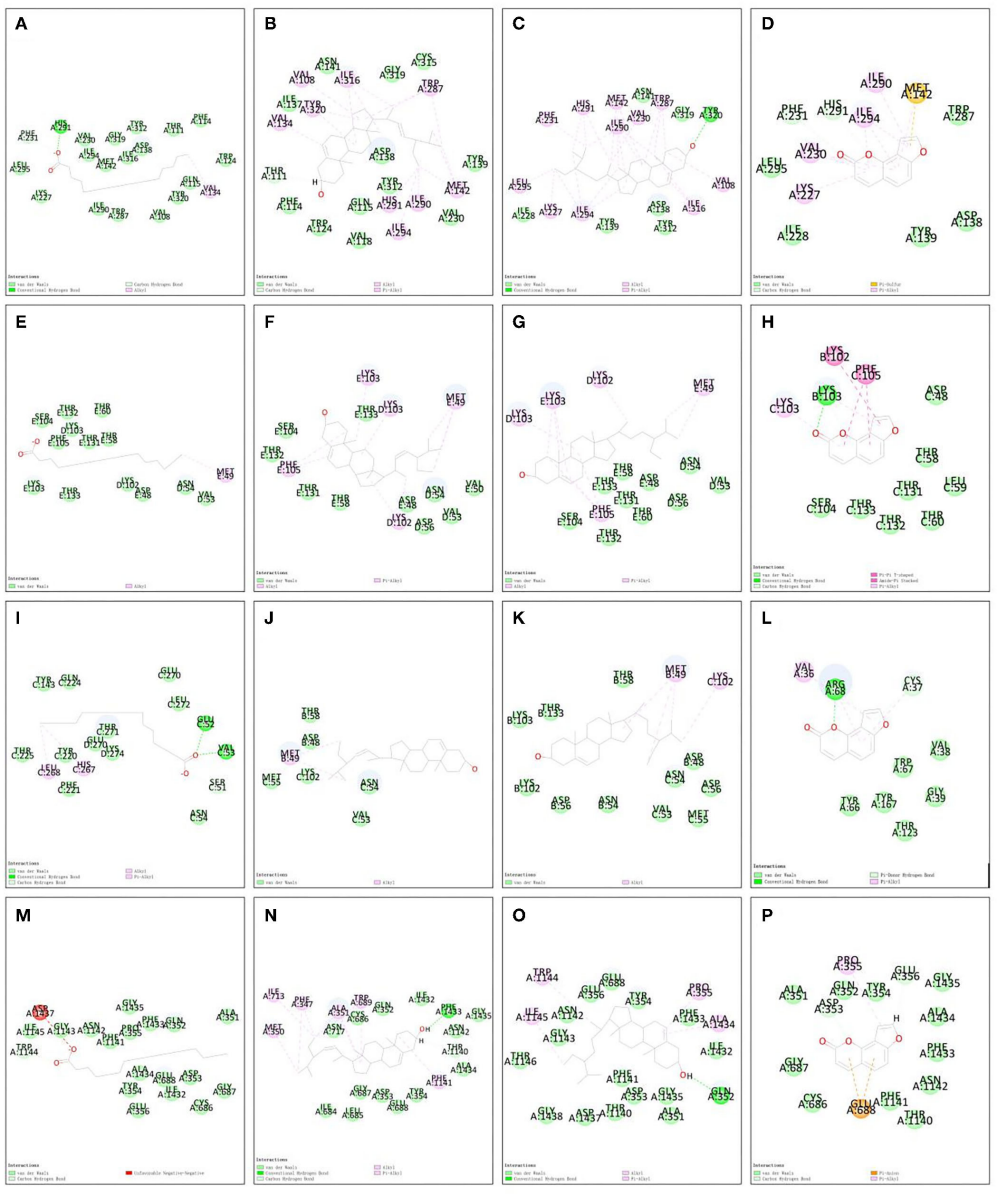
Molecular docking 2D models. **(A)** OPRK1-hexadecanoic acid complex. **(B)** OPRK1-stigmasterol complex. **(C)** OPRK1-beta-sitosterol complex. **(D)** OPRK1-angelicin complex. **(E)** GABRA5-hexadecanoic acid complex. **(F)** GABRA5-stigmasterol complex. **(G)** GABRA5-beta-sitosterol complex. **(H)** GABRA5-angelicin complex. **(I)** GABRP-hexadecanoic acid complex. **(J)** GABRP-stigmasterol complex. **(K)** GABRP-beta-sitosterol complex. **(L)** GABRP-angelicin complex. **(M)** SCN3B-hexadecanoic acid complex. **(N)** SCN3B-stigmasterol complex. **(O)** SCN3B-beta-sitosterol complex. **(P)** SCN3B-angelicin complex.

The 3D structures of hexadecanoic acid, stigmasterol, beta-sitosterol, and angelicin were drawn based on their structural formulae using Chem3D software. The 3D structures of the core proteins OPRK1, GABRA5, GABRP, and SCN3B were downloaded from the PDB database and exported to PDB format. The Vina script was used to calculate the binding energy of ligands and receptors, as shown in Table 1. GABRA5, GABRP, and SCN3B were all unable to form a stable docking model with hexadecanoic acid due to binding energies >−5.0 kcal/mol. However, the binding energies of the remaining dockers were all lower than −5.0 kcal/mol, indicating stable docking. In addition, the active molecules and corresponding target proteins were docked using the Discovery Studio 2019 software, and the LibDockScore was calculated, as shown in Table 1. The results suggested that the active ingredients hexadecanoic acid, stigmasterol, beta-sitosterol, and angelicin were able to dock semi-flexibly with the core proteins OPRK1, GABRA5, GABRP, and SCN3B, revealing the docking sites. The docking model LibDockScore of core protein OPRK1 with active ingredients hexadecanoic acid, stigmasterol, and beta-sitosterol, core protein GABRA5 with active ingredient beta-sitosterol, and core protein SCN3B with active ingredients stigmasterol and beta-sitosterol were all >100 (Figures 7, 8). The dimer formed by active ingredient stigmasterol and core protein OPRK1 was the most stable in terms of Root Mean Square Deviation (RMSD), chemical energy, and docking fraction, while the dimer formed by active ingredient beta-sitosterol and core protein SCN3B was the second most stable. Finally, the results of the compounds exported by Vina and the three-dimensional and 2-dimensional molecular docking presentations with protein ligands were imported into Pymol using Discovery Studio 2019 software (Figures 7, 8). In this study, it was demonstrated that the beneficial effects of Shunaoxin pill on diabetic cognitive impairment are mediated by the active ingredients hexadecanoic acid, stigmasterol, beta-sitosterol, and angelicin on the diabetic cognitive impairment-related proteins OPRK1, GABRA5, GABRP, and SCN3B.

**Table 1:** Results of simulated molecular docking by Autodock-Vina, Discovery Studio 2019.

**Protein**	**Compound**	**Free energy of Vina** **(kcal·mol^−1^)**	**RMSD**	**DS(LibDockScore)**	**Hydrogen bond interaction**	**Hydrophobic interaction**
OPRK1 (4DJH)	Hexadecanoic acid	−5.0	2.040	107.731	HIS:291, PHE:231	VAL:134
OPRK1 (4DJH)	Stigmasterol	−9.0	2.295	120.908	THR:111	TRP:287, ILE:316, VAL:108, TYR:320, VAL:134, HIS:291, ILE:294, ILE:290, MET:142
OPRK1 (4DJH)	Beta-sitosterol	−8.1	2.378	118.529	TYR:320	VAL:108,ILE:316, ILE:294, LYS:227, LEU:295, PHE:231, HIS:291, MET:142, ILE:290, VAL:230, TRP:287
OPRK1 (4DJH)	Angelicin	−6.9	2.207	87.6618	HIS:291, PHE:231	MET:142, ILE:290, ILE:291, VAL:230, LYS:227
GABRA5 (5O8F)	Hexadecanoic acid	−2.9	2.157	88.3026	–	MET:49
GABRA5 (5O8F)	Stigmasterol	−5.7	3.447	96.615	–	MET:49, LYS:103, PHE:105, LYS:102
GABRA5 (5O8F)	beta-sitosterol	−5.0	3.624	102.021	–	PHE:105, MET:49, LYS:102, LYS:103
GABRA5 (5O8F)	Angelicin	−5.5	1.056	68.2034	LYS:103	PHE:105, LYS:102, LYS:103
GABRP (4COF)	Hexadecanoic acid	−4.9	1.288	71.5379	GLU:52, VAL:53, SER:51	HIS:267, LEU:268
GABRP (4COF)	Stigmasterol	−7.9	28.196	34.1934	–	MET:49
GABRP (4COF)	Beta-sitosterol	−5.8	2.202	44.4127	–	LYS:102, MET:49
GABRP (4COF)	Angelicin	−8.0	1.509	57.8331	ARG:68, CYS37	VAL:36
SCN3B (7TJ8)	Hexadecanoic acid	−4.6	1.587	90.199	TRP:144	ASP:1437
SCN3B (7TJ8)	Stigmasterol	−8.9	1.844	111.354	PHE:1433, THR:1140	PHE:1141, TRP:689, ALA:351,PHE:347, ILE:713, MET:350
SCN3B (7TJ8)	Beta-sitosterol	−9.5	2.334	110.397	GLN:352	ALA:1434, PRO:355, TRP:1144, ILE:1145
SCN3B (7TJ8)	Angelicin	−6.9	13.405	64.1714	GLU:356, ASP:353	GLU:688, PRO:355

## Discussion

In this study, 18 potential targets of the Shunaoxin pill were obtained, and 16 potential core target genes were identified. Subsequently, a Shunaoxin pill-disease-target gene PPI network was constructed. The core proteins identified in PPI were OPRK1, GABRA5, GABRP, and SCN3B.

The OPRK1 gene encodes an opioid receptor, and methylation of the gene has been linked to the development of Alzheimer's disease. It is considered a drug target for the treatment of neurological diseases, playing an essential role in the development of cognitive impairment (57). The opioid receptors are mainly located in the hippocampal region, where neuroprotective effects reduce beta-amyloid production (58–60). Hiramatsu et al. reported that in a mouse model, OPRK1 agonists slowed the progression of cognitive dysfunction due to Aβ deposition (61). Diabetes can lead to massive production of islet amyloid polypeptide (IAPP), which crosses the blood-brain barrier and is deposited in the brain (hippocampus), further causing misfolding and aggregation of β-amyloid, resulting in cognitive impairment (62, 63). Hippocampal volume atrophy is observed in patients with type 2 diabetes who develop cognitive impairment (18, 42). Thus, the OPRK1 gene may be involved in the development of cognitive impairment in diabetes by affecting β-amyloid. In the present study, the Shunaoxin pill active ingredient stigmasterol was found to bind the most stably with the core protein OPRK1 in T2DM-induced cognitive impairment. This further confirms that OPRK1 plays a vital role in diabetic cognitive impairment.

GABRA5 encodes a receptor for the α5 subunit of GABA and is highly expressed in pyramidal neurons of the hippocampus (64). Furthermore, GABRA5 gene expression levels were correlated with memory function and learning index (65). In aged rats, GABRA5-encoded receptor protein expression was also associated with cognitive performance, spatial working memory, and neuronal apoptosis in aged rats (66). Moreover, it is involved in the neurophysiological features of cognitive decline in rats and humans (65). Although the exact mechanism is unknown, cognitive deficits are mainly associated with hippocampal damage in T2DM (67). In this study, GABRA5 was found to be involved in diabetes-related cognitive impairment as one of the core target proteins of the Shunaoxin pill, which may be related to its high expression in the hippocampus.

GABRP is the π-subunit of the amino acid-like inhibitory neurotransmitter gamma-aminobutyric acid (GABA) A receptor (68). GABRP was found to improve glucose tolerance and increase insulin sensitivity in the peripheral tissues of diabetic mice, interacting with GABA to maintain normal metabolism and blood glucose stability (69, 70). This study identified GABRP as one of the core target proteins of the Shunaoxin pill for preventing diabetes-related cognitive impairment. Brain insulin resistance and disorders of intracellular glucose metabolism are associated with abnormal glucose transport in diabetes (71). Brain insulin resistance in the brain may fail to stimulate the clearance of Aβ. Accumulating Aβ in neurons leads to neurodegeneration or neuronal loss, which causes cognitive impairment (72). According to previous studies, GABRP may protect against diabetes-related cognitive impairment by maintaining normal metabolism and glucose stability.

The sodium channel β3 subunit (SCN3B) is an ion channel gene that is upregulated in the dorsal root ganglion during nerve injury, suggesting neuropathic injury (73). Stimulation of peripheral nerves in diabetic peripheral neuropathy (DPN) patients was demonstrated to activate cognitive-related areas of the brain, such as the temporal lobe and hippocampus (74). Furthermore, the reduced sensory conduction velocity of the peroneal nerve is often seen in DPN. In the present study, beta-sitosterol, an active ingredient in the Shunaoxin pill, was shown to bind stably to SCN3B, thus possibly playing a role in the development of diabetes-induced cognitive impairment. Therefore, we hypothesize that its mechanism of action on cognition may involve neurotransmission sodium channels.

The findings of this study provide a foundation for further exploration of TCM therapeutic targets for diabetic cognitive impairment. Network pharmacology revealed that the active ingredients in the Shunaoxin pill—hexadecanoic acid, stigmasterol, beta-sitosterol, and angelicin—could be semi-flexibly docked to the receptor-ligands of their respective core proteins, OPRK1, GABRA5, GABRP, and SCN3B. The active ingredient stigmasterol formed the most stable dimer with the core protein OPRK1, and the active ingredient beta-sitosterol formed the second most stable dimer with the core protein SCN3B. The present study illustrates the action of the active ingredients—hexadecanoic acid, stigmasterol, beta-sitosterol, and angelicin—on the diabetic cognitive impairment-related proteins OPRK1, GABRA5, GABRP, and SCN3B that are responsible for the beneficial properties of the Shunaoxin pill on cognitive impairment in diabetic patients.

However, this study also has some limitations. This study is based on network pharmacology and lacks experiments exploring the specific mechanism of the Shunaoxin pill's active ingredients targeting core proteins in diabetic cognitive impairment. Further validation of core proteins in clinical samples of the disease should be performed. Since cognitive impairment includes multiple disorders, this study focused only on AD-related datasets; future studies should explore more cognitive impairment-related datasets.

## Conclusion

This study demonstrated that the Shunaoxin pill is pharmacologically effective for cognitive impairment in diabetic patients. Its active ingredients, hexadecanoic acid, stigmasterol, beta-sitosterol, and angelicin, target the proteins OPRK1, GABRA5, GABRP, and SCN3B associated with diabetic cognitive impairment. This research provides a foundation for further exploration of TCM therapeutic targets for cognitive impairment in diabetic patients.

## Data availability statement

The original contributions presented in the study are included in the article/supplementary materials, further inquiries can be directed to the corresponding author.

## Author contributions

YG and NL proposed and designed the study and collected and analyzed the data. YG provided the analysis tools and performed quality control. YG, NL, and XK wrote the manuscript. All authors contributed to the article and approved the submitted version.

## Conflict of interest

The authors declare that the research was conducted in the absence of any commercial or financial relationships that could be construed as a potential conflict of interest.

## Publisher's note

All claims expressed in this article are solely those of the authors and do not necessarily represent those of their affiliated organizations, or those of the publisher, the editors and the reviewers. Any product that may be evaluated in this article, or claim that may be made by its manufacturer, is not guaranteed or endorsed by the publisher.

## Abbreviations

DM: diabetes mellitus
T2DM: type 2 diabetes mellitus
SNX: Shunaoxin dropping pills
OPRK1: opioid receptor kappa 1
GABA: gamma-aminobutyric acid
GABRA5: gamma-aminobutyric acid type A receptor subunit alpha5
GABRP: gamma-aminobutyric acid type A receptor subunit pi
SCN3B: sodium voltage-gated channel beta subunit 3.
